# Key Genes Associated with Tumor-Infiltrating Non-regulatory CD4- and CD8-Positive T Cells in Microenvironment of Hepatocellular Carcinoma

**DOI:** 10.1007/s10528-021-10175-3

**Published:** 2022-01-29

**Authors:** Zijun Zhao, Chaonan Wang, Peishan Chu, Xin Lu

**Affiliations:** 1 Department of Liver Surgery, Peking Union Medical College Hospital, Chinese Academy of Medical Sciences and Peking Union Medical College, 1 Shuaifuyuan, Wangfujing, Beijing, 100730 China; 2 Department of Vascular Surgery, Peking Union Medical College Hospital, Chinese Academy of Medical Sciences and Peking Union Medical College, Beijing, China; 3 Department of Cardiac Surgery, Peking Union Medical College Hospital, Chinese Academy of Medical Sciences and Peking Union Medical College, Beijing, China

**Keywords:** Hepatocellular carcinoma, Genes, Tumor-infiltrating T cell, Tumor microenvironment, Prognosis

## Abstract

The immune microenvironment in hepatocellular carcinoma (HCC), especially T-cell infiltration, plays a key role in the prognosis and drug sensitivity of HCC. Our study aimed to analyze genes related to non-regulatory CD4^+^ and CD8^+^ T cell in HCC. Data of HCC samples were downloaded from The Cancer Genome Atlas (TCGA) database. According to stromal and immune score retrieved by Estimation of Stromal and Immune cells in Malignant Tumor tissues using Expression data (ESTIMATE) algorithm, differentiated expressed genes (DEGs) between high and low stromal/immune scoring groups were collected. Using Cibersort algorithm, abundance of immune cells was calculated and genes related with CD4^+^ and CD8^+^ T cells were selected. Protein–protein interaction (PPI) networks and networks of microRNA (miRNA)–target gene interactions were illustrated, in which CD4^+^ and CD8^+^ T cell-related core genes were selected. Finally, Cox regression test and Kaplan–Meier (K–M) survival analysis were conducted. Totally, 1579 DEGs were identified, where 103 genes and 407 genes related with CD4^+^ and CD8^+^ T cell were selected, respectively. Each of 30 core genes related to CD4^+^ T cells and CD8^+^ T cells were selected by PPI network. Four genes each related with the two types of T cells had a significant impact on prognosis of HCC patients. Amongst, KLRB1 and IL18RAP were final two genes related to both two kinds of T cells and associated with overall survival of the HCC patients.

**Supplementary Information:**

The online version contains supplementary material available at 10.1007/s10528-021-10175-3.

## Introduction

Primary liver cancer (PLC) is the third most common cause of cancer-related mortality (8.3%) and the sixth most commonly diagnosed cancer (4.7%) worldwide in 2020 (Sung et al. 2020). Hepatocellular carcinoma (HCC) is the dominant type, accounting for 75% to 85% of all cases of PLC. Surgery is the major treatment for HCC, including local resection and liver transplantation (Galle et al. 2017; Weinmann and Galle 2020). For advanced stage and inoperable patients, however, the recommended treatment plans include systemic therapy with first and second line of multi-kinase inhibitor, such as sorafenib and regorafenib (Bruix et al. 2017; Llovet et al. 2008). Although great progress has been made in diagnostics and therapy during the past decades, HCC remains a terminal disease due to drug resistance and distant metastasis, with a 5-year survival of 18.1% across all stages and 2.3% for advanced disease. For patients who suffered from advanced HCC and for whom systemic therapy is the only choice, the 1-year survival rate has been less than 50% since diagnosis is confirmed (Jemal et al. 2017; Giannini et al. 2015).

In recent years, tumor microenvironment (TME) has been paid much attention by community of medical oncology (Hanahan and Weinberg 2011). Basically, TME consists of extracellular matrix and variety of cells, including fibrocytes, cells derived from vascular structure, and immune cells (Baghban et al. 2020). For liver, it also has various types of cells. They can be classified as two types: one is hepatocellular cell (liver parenchymal cell) and non-parenchymal cells [liver sinusoidal endothelial cells, Kupffer cells, hepatic stellate cells, liver natural killer (NK) cell, DC cell, T/B cell, etc.] (Jiang et al. 2020). These cells together build a complex TME in liver and regulate tumor cell biological activities in different ways. The immune compartment in TME of liver is called as tumor-infiltrating lymphocytes (TILs), which is thought to be an innate immune weapon for people to eliminate cancerous cells. CD4^+^ and CD8^+^ T cells are predominant in TILs and early clinical studies found that high level of T-cell infiltration in TME results in a lower recurrence rate and a better survival for patients with HCC (Unitt et al. 2006; Cariani et al. 2012). 18 years ago, Japanese scientists invented a novel algorithm to calculate the ratio of immune cells and stromal cells in malignant tumors via gene expression signature derived from The Cancer Genome Atlas (TCGA). It is called Estimation of Stromal and Immune cells in Malignant Tumor tissues using Expression data (ESTIMATE) algorithm. This algorithm can evaluate and predict the purity of certain kind of tumor (Yoshihara et al. 2013). Numerous clinicians and bioinformaticians have extracted data and initiated research of varieties of solid tumors. Xiang et al. found that increased stromal, immune, and Estimate scores were significantly correlated with better prognosis of HCC patients (Xiang et al. 2021), which is also found in research of other clinical teams (Yu et al. 2021; He et al. 2020a). Nevertheless, existence of T cells cannot avoid tumorigenesis because they can express some immune inhibitory molecules. Activated T cells can express immunosuppressive receptors, such as programmed cell death protein 1 (PD-1) and cytotoxic T lymphocyte-associated protein 4 (CTLA-4). When stimulated effector T cells attack tumor cells, the immune response is strong at the early time and then turns weaker due to the interactions between PD-1 and its ligands PD-L1 expressed on tumor cells. Then the tumor cells get tolerated to the initial immune response and apoptosis, exhaustion, and phenotype conversion of T cell occurs (Kudo 2019; Alsaab et al. 2017). Different from PD-1, CTLA-4 expressed on matured T cells combines with its ligand CD80/86 on various types of antigen-presenting cells, which can also suppress the immune response initiated by interactions between CD28 of T cells and CD80/86.

The liver is a ‘tolerogenic’ organ for it is an ideal organ for transplantation surgery and liver allograft is more tolerogenic than that from other organs (Jiang et al. 2020; Zimmermann et al. 1984). This trait of liver tissue is due to the immunosuppressive molecules and cells in liver TME. This immunosuppressive characteristic in liver tissue, however, can also cause immune invasion, which is an essential trigger for initiation and development of HCC (Shlomai et al. 2014). The level of immunosuppression in liver TME is closely related to prognosis of HCC patients. Hence, to reconstruct a strong anti-tumor immunity, the immune landscape of HCC and detailed information of some important immune cells are necessary to be investigated (Fu et al. 2019). In this study, we investigated the gene expression pattern associated with tumor-infiltrating non-regulatory CD4^+^ and CD8^+^ T cells of HCC. The results are expected to provide therapeutic targets for future immunotherapy of HCC.

## Materials and Methods

### Data Acquisition

The expression data, RNA-sequencing fragments per kilobase of transcript per million mapped reads, and corresponding clinical information from The Cancer Genome Atlas Hepatocellular Carcinoma (TCGA-LIHC) data collection were collected from TCGA GDC database (https://portal.gdc.cancer.gov/). Data acquisition was on 06-03-2021.

### Differentially Expressed Genes (DEGs) Identification

We first transferred and made annotations by Perl (Version 5.30.0) for the probes from expression data according to the annotation files downloaded from Ensemble genome browser 103 (http://ftp.ensembl.org/pub/current_gtf/homo_sapiens/). R studio based on R (v4.0.4) was used to arrange the expression data. R package “limma” (Version 3.46.0) was used. The items in which multiple probes matched to the same gene were filtered and the gene expression was calculated as mean value of all these probes. Stromal scores and immune scores of 374 tumor tissue were calculated using the ESTIMATE package (Version 1.0.13). According to the median level of the stromal/immune score, the DEGs between high stromal/immune scores and low stromal/immune scores were screened using the “limma” packaged with screening criteria of |log fold change (FC)|> 1 and false discovery rate (FDR) < 0.05. Then we plotted a heatmap including top 50 up-regulated DEGs and top 50 down-regulated DEGs according to stromal score and immune score, respectively. The package “pheatmap” (Version 1.0.12) was used. Using R package “VennDiagram” (Version 1.6.20), we used two Venn diagrams to illustrate overlapping DEGs of up-regulated and down-regulated genes according to both immune and stromal scores. The overlapping DEGs of up- and down-regulated genes were used in the following analysis.

### Functional Enrichment Analysis

Using R package “org.Hs.eg.db” (Version 3.12.0), the ensemble ID of DEGs of both up- and down-regulated genes were retrieved. The “biological process” (BP), “cellular components” (CC), and “molecular functions” (MF) terms of the Gene Ontology (GO) annotation and the Kyoto Encyclopedia of Genes and Genomes (KEGG) pathways were analyzed via R package of “org.Hs.eg.db,” “clusterProfiler” (Version 3.18.1), “enrichplot” (Version 1.10.2), and “ggplot2” (Version 3.3.3) to investigate the functions of the up-regulated and down-regulated overlapping DEGs. The terms with *P* < 0.05 were considered to be significant results.

### Screening of CD4^+^ and CD8^+^ T Cell-Related Genes

Matrix of reference gene expression in 22 kinds of immune cells was downloaded from CIBERSORTx (https://cibersortx.stanford.edu/) as the reference file (Newman et al. 2015, 2019). Based on the Cibersort algorithm and “limma” package, the relative abundance (%) of 22 tumor-infiltrating immune cells in selected HCC samples were estimated (Newman et al. 2015). After that, the correlation coefficient (*r*) between overlapping DEGs and infiltrating abundance of immune cells (CD4^+^ and CD8^+^ T cells) was calculated using the Spearman correlation test. Selected CD4^+^ and CD8^+^ T cell-related genes were identified with correlation coefficient |*r*|> 0.23.

### Construction of the Protein–Protein Interaction (PPI) Network and Core Genes Selection

The interactions between CD4^+^ and CD8^+^ T cell-related genes were retrieved from the PPI website STRING database (https://www.string-db.org/) with a PPI score setting as 0.35 and 0.90, respectively. The PPI network visualization was performed by Cytoscape software (version: 3.6.0). Then Perl language and R were used to summary the top 30 core genes in retrieved PPI network of CD4^+^ and CD8^+^ T cell, respectively.

### Construction of the MicroRNA (miRNA)–Target Gene Networks

To predict miRNA–target gene interactions, core genes in PPI networks of CD4^+^ and CD8^+^ T cell were input to miRwalk 3.0 (http://mirwalk.umm.uni-heidelberg.de/), a miRNA–target gene prediction database. For settings of parameters, the miRNA–target gene interactions with a score > 0.95 and those that existed in both the TargetScan and miRDB databases were selected. The miRNA–target gene network for both CD4^+^ and CD8^+^ cells were visualized in Cytoscape.

### Survival Analysis

The clinical data and expression data of CD4^+^ and CD8^+^ T cell-related genes were integrated using Perl language and “limma” package. The prognosis-related genes were summarized by function “coxph()” and demonstrated with forest plots. The overall survival (OS) and OS status in the clinical data were used to perform survival analysis via R package “survival” (Version 3.2-7) and “survminer” (Version 0.4.8). In this process, the immune cell-related genes were divided into low-expression group and high-expression group based on the median level of gene expression, together with a log-rank statistical test. The cut-off was set as a *P* value < 0.05 to select the significantly prognosis-related genes in CD4^+^ and CD8^+^ group and then Kaplan–Meier (K–M) survival curves were plotted.

## Results

### Differentiated Expression Gene was Collected Based on Stromal Scores

The total number of samples in TCGA-LIHC data is 424, 374 of which were samples of HCC, while the other 50 samples were normal liver tissue. Via ESTIMATE algorithm, stromal scores and immune scores were successfully calculated in all 374 HCC samples. The higher the stromal/immune scores were, the higher proportions of stromal/immune cells in the samples were. According to the stromal scores in all HCC samples, the median score was − 659.4628. The number of samples whose scores higher than the median level was 187, the same as that of samples with scores no more than the median level. The gene expression data of 374 HCC samples have been further processed by removing replicated genes and genes with an average expression no more than 0.1. Through calculation of difference of gene expression, final 1891 genes were defined as DEGs. Among, 1670 genes were up-regulated in samples with a higher stromal score, whereas 221 genes were down-regulated in samples with a higher stromal score. According to the value of logarithm of FCs (logFC) of genes, top 50 up-regulated and top 50 down-regulated genes are plotted in the heatmap (Supplementary Fig. 1) and summarized in Supplementary Table 1.

### Differentially Expressed Genes were Collected Based on Immune Scores

According to the calculation of immune scores in 374 HCC samples, the median value was 432.3971. The number of samples with scores no more than median level equaled to that of samples with scores exceeding the median value (187 for each group). Based on the selection criteria aforementioned, final 1579 genes were designated as DEGs. Among, 1418 genes were up-regulated in samples with immune scores higher than median level, while 161 genes were down-regulated in samples whose immune scores were less than median value. The top 50 up-regulated and top 50 down-regulated genes are illustrated in heatmap (Supplementary Fig. 2) and the information of these genes are summarized in Supplementary Table 2.

### Overlapping Up-regulated and Down-regulated Genes According to Both Stromal and Immune Scores were Analyzed in GO and KEGG Database

According to the results mentioned above, genes which were up-regulated based on both stromal and immune scores and which were down-regulated are collected and illustrated in Venn diagrams (Supplementary Fig. 3a, b). The number of overlapping up-regulated DEGs was 1076 and the number of overlapping down-regulated DEGs was 62. Table 1 summarizes the top 50 overlapping up-regulated–down-regulated DEGs.

**Table 1:** Overlapping up-regulated and down-regulated DEGs based on both stromal and immune scores

Up-regulated gene	logFC	Down-regulated gene	logFC
TRARG1	7.666054729	KLK4	− 2.890539921
CRTAC1	5.532685203	RGSL1	− 2.205440106
IGFN1	5.265016334	RHBDL3	− 2.184229516
C16orf89	4.865872612	LINC02587	− 2.144128848
IGKV1D-42	4.825363156	AP000593.3	− 2.023942443
IGLV5-45	4.512147135	AURKBP1	− 1.894678211
IGHV1-58	4.441232443	AC007277.1	− 1.891447694
IGLV10-54	4.440730135	AL590483.2	− 1.881354742
IGKV2D-40	4.432250477	C1QTNF3	− 1.860001267
IGKV6-21	4.431686791	RASL10B	− 1.791626005
IL11	4.009377903	RHBG	− 1.780671072
OMG	3.9775617	PAGE4	− 1.778360546
IGHV3-73	3.931511939	AC104088.1	− 1.750003438
CCL19	3.858908479	ACTN2	− 1.726351182
IGLV3-16	3.791071906	CTNNA2	− 1.717561344
CHIT1	3.764407355	KCNU1	− 1.691739858
CHRNA1	3.747690595	AC026765.3	− 1.685375046
IGHV1-2	3.724027271	AC011747.1	− 1.585204355
CR2	3.71985571	NOTUM	− 1.557252788
IGLC7	3.717952082	LGR5	− 1.556438279
IGLV2-8	3.714584762	AC069294.1	− 1.541662443
IGKV3D-15	3.64296302	AL163953.1	− 1.505394178
IGLV4-69	3.61053471	ABHD1	− 1.485544254
IGHV3-11	3.574892854	LINC01970	− 1.484943844
CRYBB1	3.543085965	GLUL	− 1.445004555
TNNT3	3.507231587	GLULP4	− 1.409807598
IGHV3-64	3.45118729	MIR325HG	− 1.405286019
IGHV3-71	3.444000853	ACSL6	− 1.401318749
IGLV8-61	3.440634419	DNAJB3	− 1.394672128
DES	3.423964824	SP5	− 1.359215973
AC135068.1	3.417111295	LINC01124	− 1.341499193
IGHV3-15	3.40543775	AC010501.2	− 1.332176671
IGKV1-9	3.361738473	AC114947.1	− 1.331232992
IGHV3-33	3.349048911	AC113404.1	− 1.328616526
IGHV1-69	3.328530619	GREB1	− 1.31633006
IGHV2-70	3.312158823	MTND4LP30	− 1.316294715
IGKV1D-13	3.296648953	AC008549.1	− 1.303165529
WNT2	3.288090828	AC007406.2	− 1.2826021
IGHV3-19	3.277470772	AC013244.1	− 1.268139454
IGHV2-70D	3.276646515	SRARP	− 1.259680291
IGHV1-24	3.275489277	C5orf66	− 1.258706959
IGHV2-26	3.264057216	ASB4	− 1.251581711
IGKV3D-11	3.239636373	TBX3	− 1.243487135
PLA2G2D	3.225196379	RNU6-46P	− 1.237461012
MS4A1	3.223865765	AC005841.1	− 1.227513059
IGHG2	3.214812763	FAM169A	− 1.217327844
NDNF	3.203978929	TECTB	− 1.21055518
IGLV2-11	3.199379915	H4C10P	− 1.198960549
IGKV2-30	3.191285367	ACTG1P25	− 1.197484908
IGLV1-40	3.189484969	AC010531.5	− 1.19431992

*DEGs* differentially expressed genes, *FC* fold change

The logFC value in this table was calculated as the mean of the logFC based on stromal score and immune score. GO and KEGG analysis of these overlapping genes are illustrated in Supplementary Figs. 4a and b and 5a and b, respectively. In GO analysis, the top 10 highest enriched genes with functions of BP, CC, and MF were showed. In the picture of KEGG, the top 30 highest enriched gene pathways were showed. Most of them were associated with immune response.

### Genes Associated with Tumor-Infiltrating CD4^+^ and CD8^+^ T Cell were Identified by CIBERSORTx Algorithm

**Fig. 1:**
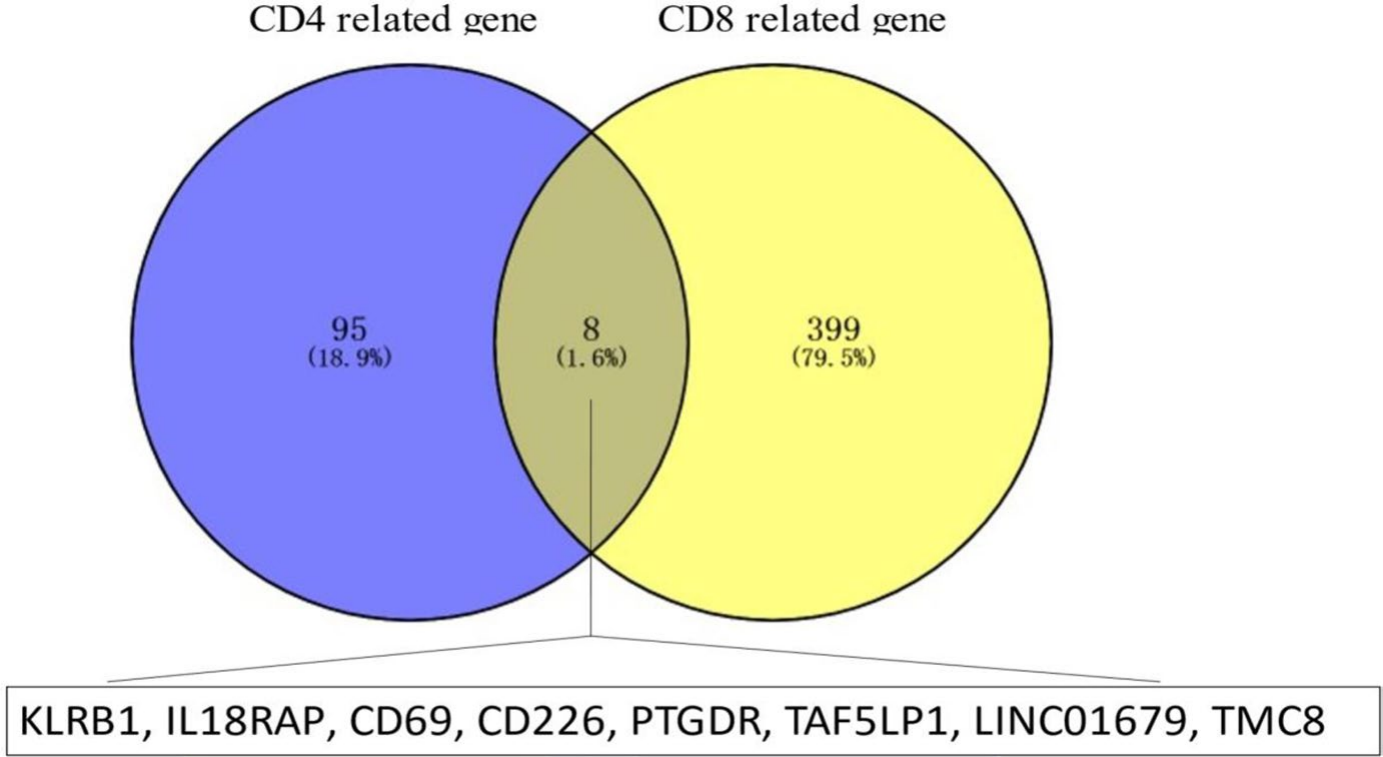
Number of CD4^+^ and CD8^+^ T cell-related genes and the gene symbols of eight overlapping genes in HCC samples. *HCC* hepatocellular carcinoma

Using Spearman’s correlation test, the correlation coefficients (*r*) of overlapping DEGs with the abundance ratio of CD4^+^ T cell and CD8^+^ T cell were successfully calculated. Genes with |*r*|> 0.23 were included. By this criteria, 103 CD4^+^ T cell-related genes and 407 CD8^+^ T cell-related genes were collected. Eight genes were found to be correlated with both kinds of T cells and this was illustrated via Venn diagram in Fig. 1. Based on the abundance ratio of several main immune cells via Cibersort algorithm, barplots of the proportion of these immune cells are demonstrated in Supplementary Fig. 6. In this picture, it is found that CD4^+^ and CD8^+^ T cells taking up a large proportion in tumor-infiltrating immune cells in TME of HCC. In this plot, only samples with *P*-value < 0.05 will be included to be used for analysis.

### Core Genes Related to Tumor-Infiltrating CD4^+^ and CD8^+^ T Cell in PPI Network were Retrieved

**Fig. 2:**
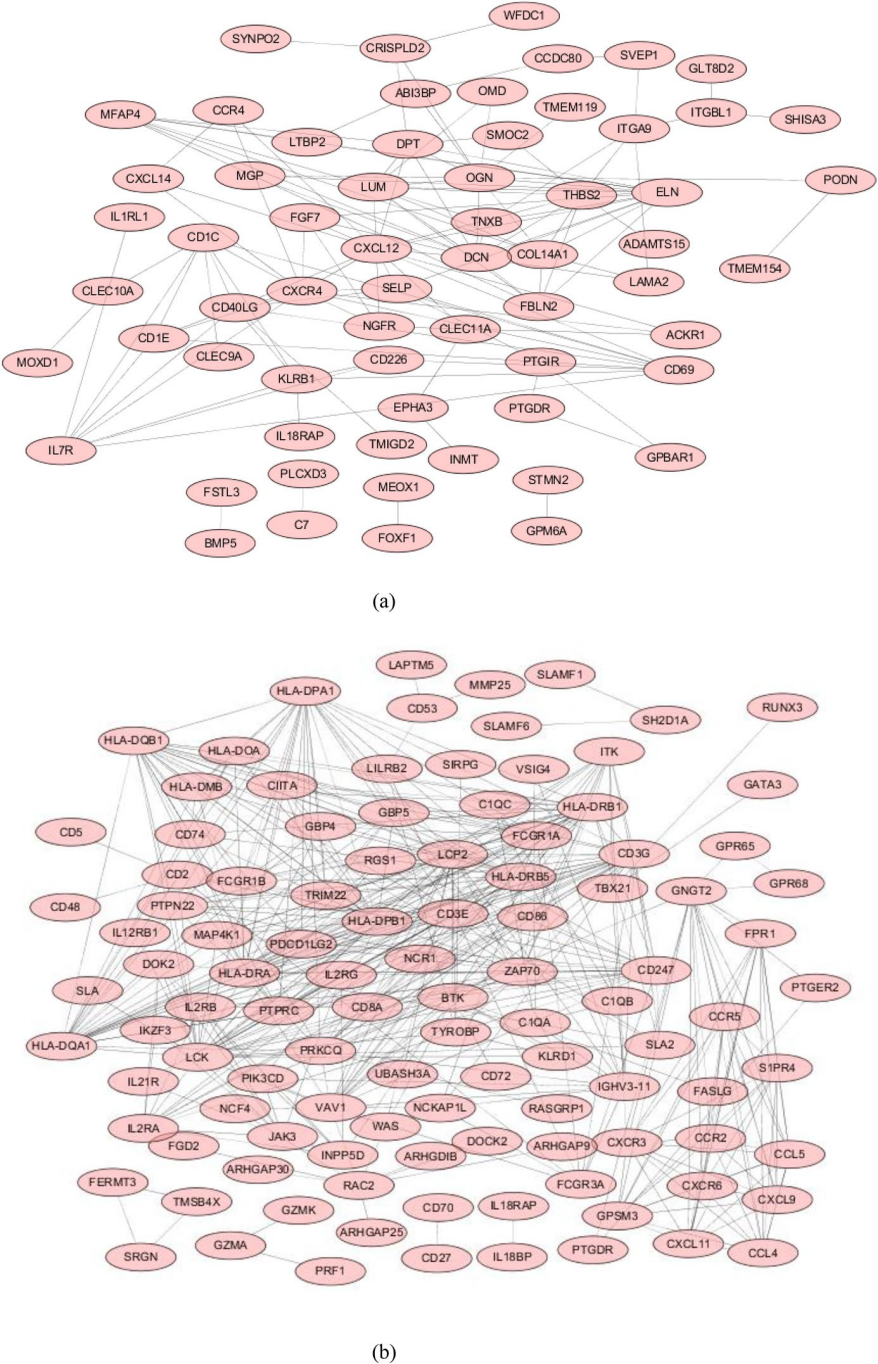
Protein–protein network of CD4^+^ T cell-related genes (**a**) and CD8^+^ T cell-related genes (**b**)

**Fig. 3:**
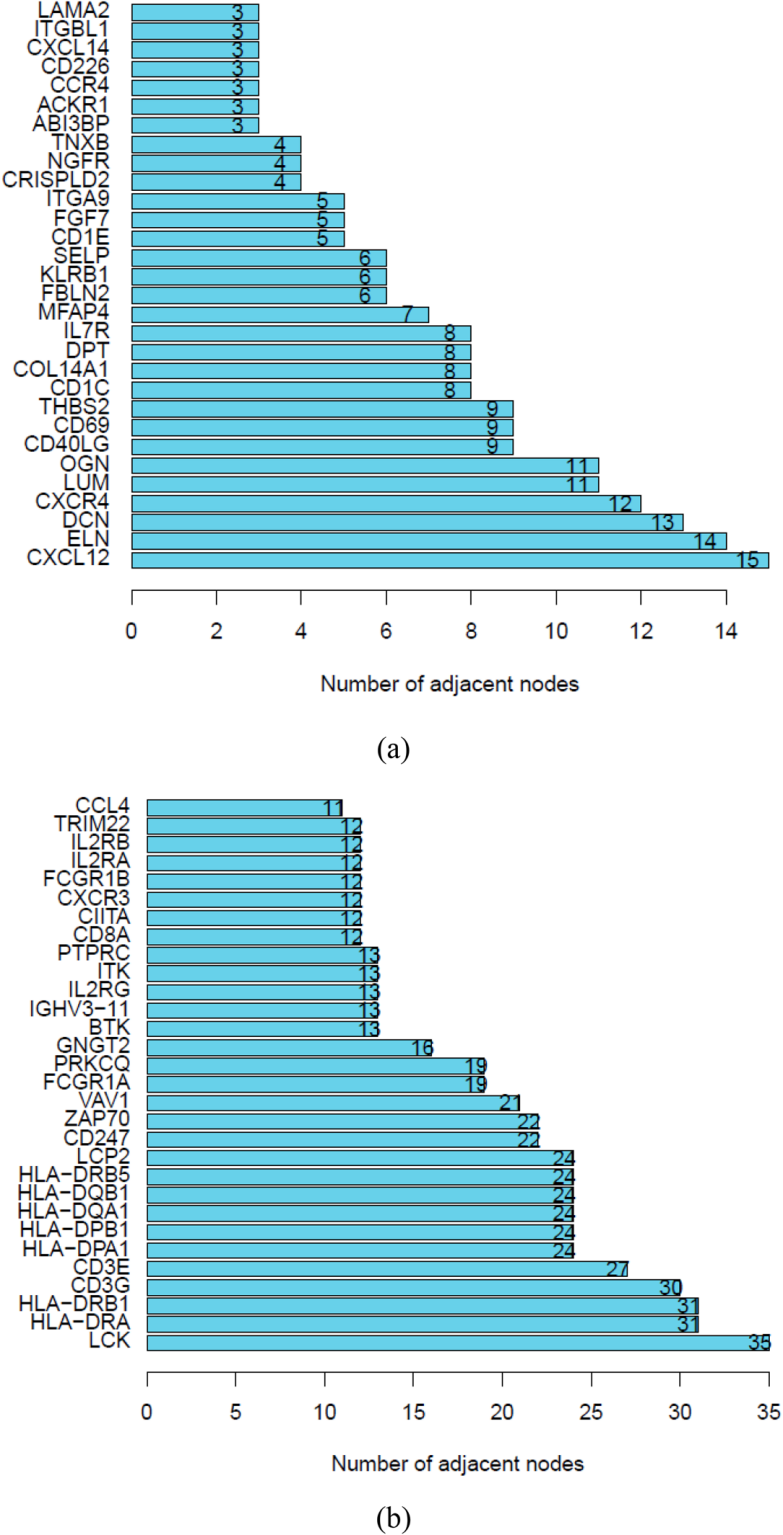
Core genes in PPI networks of T cell-related genes. **a** Collected top 30 core genes related to CD4^+^ T cells and **b** demonstrated top 30 genes associated with CD8^+^ T cells. *PPI* protein–protein interactions

Based on results of CD4^+^ and CD8^+^ T cell-related genes, PPI networks of these genes were plotted, which are shown in Fig. 2a and b. Considering the number of genes and the effect of visualization of the network, the interaction confidence of CD4^+^ T cell-related genes was set as 0.35, while that of CD8^+^ T cell-related genes was set as 0.90. Of notice, all the genes visualized in these pictures were all up-regulated DEGs according to the DEG analysis. Based on PPI data, the number of interactions for each node were summarized and core genes in PPI network were defined as the top 30 CD4^+^ and CD8^+^ T cell-related genes according to the number of interactions for each node and the plot is illustrated in Fig. 3a, b.

### miRNA–Target Gene Interaction Network of Core Genes Related to Tumor-Infiltrating CD4^+^and CD8^+^ T Cell were Visualized

The prediction of miRNA–target gene interaction was conducted by software miRwalk 3.0. Networks of predicted miRNA–gene interactions with CD4^+^ T cells and CD8^+^ T cells are illustrated in Supplementary Fig. 7a and b, respectively.

### Prognosis-Related Genes Associated with Tumor-Infiltrating CD4^+^ and CD8^+^ T Cell were Found in HCC Samples

**Fig. 4:**
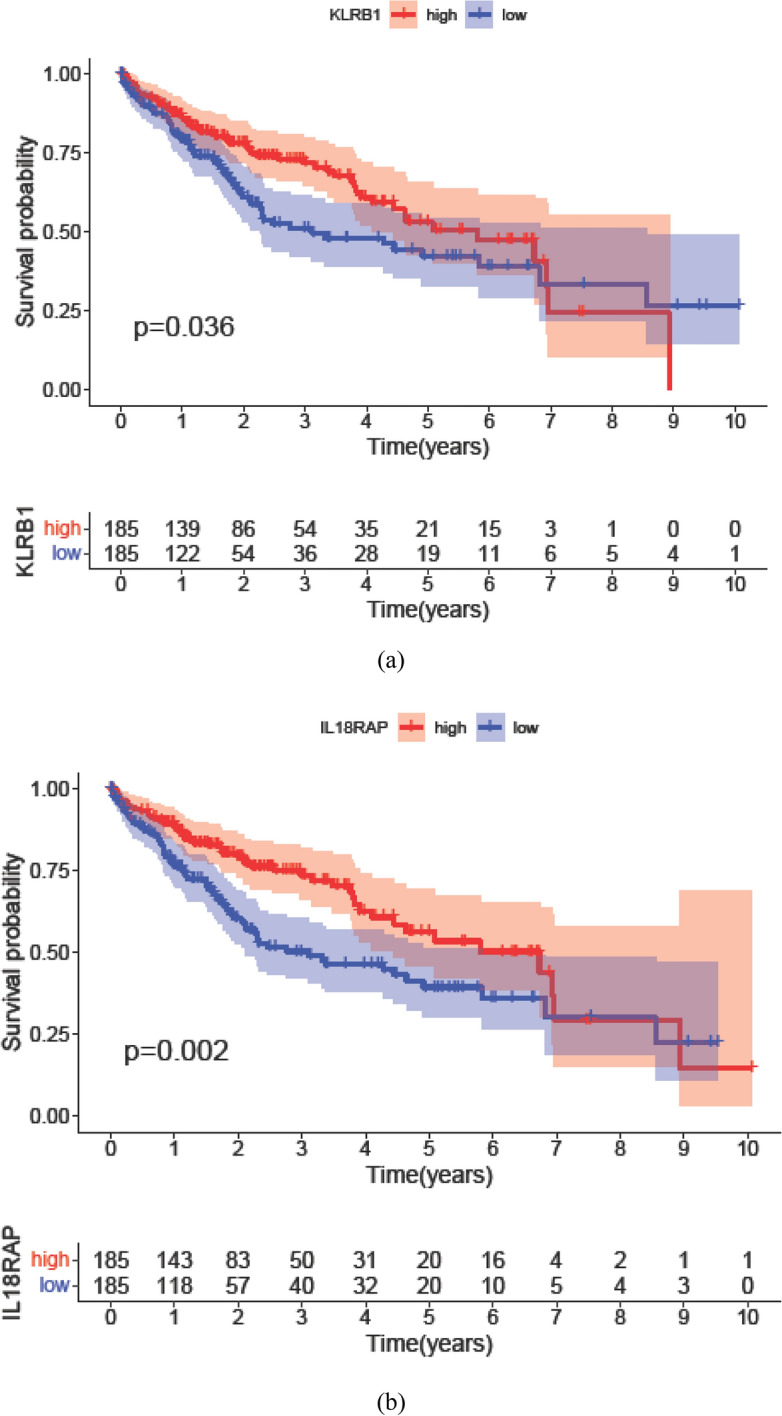
Two genes, KLRB1 (**a**) and IL18RAP (**b**) were two genes not only related to abundance of both CD4^+^ and CD8^+^ T cells but also significantly associated with prognosis of HCC patients. Both of them were beneficial to HCC patients with a HR < 1. *HCC* hepatocellular carcinoma, *HR* hazard ratio

Candidate genes for survival analysis came from CD4^+^ and CD8^+^ T cell-related genes. Cox regression method was used for OS analysis. In total, six genes of CD4^+^ T cells were found significantly related to OS of HCC patients and the forest plot has told that all these genes were beneficial to HCC patients since that the expressions of these genes increased in patients with a better prognosis [hazard ratio (HR) < 1]. Moreover, 30 genes of CD8^+^ T cell-related genes were found significantly related to OS of HCC patients. Corresponding forest plot told that 21 genes were high-risk genes whose expression increased in patients with a shorter OS (HR > 1) and the other nine genes were beneficial to HCC patients (Supplementary Fig. 8a, b). Via K–M analysis, final four genes related to CD4^+^ T cells and final four genes relate to CD8^+^ T cells were found significantly associated with prognosis (OS) of HCC patients. Among them, KLRB1 and IL18RAP were two overlapping genes not only influential to HCC patients’ OS but also related to both CD4^+^ and CD8^+^ T cell. And both of these genes were clinically beneficial to HCC patients. Their high expressions tended to result in a relatively long OS for HCC population. Survival curves of these two genes are shown in Fig. 4a and b and the other prognosis-related genes are illustrated in Supplementary Fig. 9a and b.

## Discussion

In our study, 1579 DEGs in HCC samples between high stromal/immune scores were identified, 89.8% of which were up-regulated genes and 10.2% of which were down-regulated genes. 75.9% of the whole DEGs were overlapping up-regulated genes based on both stromal and immune scores, while 38.5% were overlapping down-regulated genes. Among these overlapping DEGs, immune-related items were the major elements in top 10 enriched biological function, CC, and MFs. This suggested that DEGs played an important role in immune regulation. And this stimulated us to further explore the relationship of genes with CD4^+^ and CD8^+^ T cells. Of the 1138 overlapping DEGs, 9.1% of them were correlated with relative abundance of CD4^+^ T cells and 35.8% were associated with that of CD8^+^ T cells. Among these genes, eight of them were related with both of these two kinds of T cells and the proportion was around 1.6%. Moreover, via summarizing the immune cell proportion in different HCC samples, we found that CD4^+^ and CD8^+^ T cells were major subtypes of immune cell family, which reassured the reasonability of investigating immune-related genes. Speaking of the PPI networks, 30 core genes related to CD4^+^ T cells and 30 CD8^+^ T cell-related genes were designated. For miRNA–target networks, genes targeted by existing miRNAs took up more than 50% in two groups, which indirectly proved their core status in PPI network. In prognosis analysis, both of the Cox regression and K–M survival analysis found that only four genes related to CD4^+^ T cells and four genes correlated with CD8^+^ T cells were significantly prognosis-related factors for HCC patients. KLRB1 and IL18RAP were two overlapping genes not only related to both CD4^+^ and CD8^+^ T cells but also had a protective effect for HCC patients. Their high expression tended to appear in patients with a longer OS.

The immune tolerance and immune evasion in the liver usually causes initiation of HCC or liver metastasis from other types of solid tumors (Shlomai et al. 2014). Chronic infection of hepatitis B virus and hepatitis C virus and toxic injuries derived from alcohol and aflatoxin are typical stimulators of HCC. These risk factors are likely to induce immunosuppression through host–viral interaction or chronic inflammation (Woller et al. 2015). Briefly, elements of immunosuppression include inhibitory cytokines or chemokines, defects of antigen presentation, and immunosuppression cells in liver microenvironment (Hato et al. 2014; Zhang et al. 2010). In addition to HCC, immune tolerance can also contribute to liver metastasis of other solid malignancies. Liver metastasis tend to weaken the effect of systemic therapy, such as immune checkpoint inhibitors, including PD-1 and CTLA-4 antibodies. It is in part associated with a decreased infiltration of CD8^+^ T cell (Tumeh et al. 2017). As a result, more information of immune landscape of HCC microenvironment should be extracted and the methods to reverse the repressive and tolerant immunity of liver microenvironment are badly needed to be found.

In our study, we found that KLRB1 and IL18RAP were up-regulated in the HCC samples with a higher than median immune/stromal score and the Spearman correlation coefficient (*r*) of the two genes were all more than 0.23. Patients with a longer OS had a relatively high expression of KLRB1 and IL18RAP. Speaking of survival, higher expression of these two genes corresponds to a longer survival of patients with HCC. Several researches had found the relationship between solid tumors and these two genes.

KLRB1, whose full name is killer cell lectin-like receptor subfamily B 1 and is also named as CD161, is a member of NKRP1 subfamily which codes NKRP1A molecule (Mesci et al. 2006). This molecule is a kind of type II transmembrane glycoprotein whose molecular weight ranges between 40 and 44 kDa (Lanier et al. 1994; Poggi et al. 1996). NKRP1A has an extracellular region, including a C-type lectin domain, a transmembrane region, and an intracellular region, including an immunoreceptor tyrosine-based inhibitory motif (Lanier et al. 1994; Rosen et al. 2008). NKRP1A is mainly expressed in NK cells as well as T cells secreting IL-17, CD8^+^ T cells, and γδT cells (Poggi et al. 1998; Maggi et al. 2010; Turtle et al. 2011). Functions of NKRP1A are different in different immune cells. Scientists have found that NKRP1A can inhibit cytotoxicity and interferon-γ (IFN-γ) secretion of NK cells (Aldemir et al. 2005). Different from this, NKRP1A on T cells on one hand enhances expression of IL-17, tumor necrosis factor-α (TNF)-α, and IFN-γ and on the other hand reduces the production of TNF in CD8^+^ T cells (Rosen et al. 2008; Germain et al. 2011). Clinical scientists have also focused on this gene in studies of solid malignancies. A clinical study led by Zhang et al. found that KLRB1 was a potential prognostic marker for esophageal squamous cell carcinoma (Zhang et al. 2020). In a study led by research team from France, receptor–ligand pair of CD161–lectin-like transcript 1 (LLT) has a positive impact on survival of patients suffered from non-small cell lung carcinoma (*P* = 0.016, HR = 0.88) (Braud et al. 2018).

IL18RAP, short for interleukin 18 receptor accessory protein, expresses the accessory β-subunit of the heteromeric receptor for IL18, a cytokine regulating the immunity by its pro-inflammatory effect and promoting tumor initiation and progression (Kaplanski 2018). In depth, IL18RAP strengthens the combining force between IL18 and its receptor, which has a positive effect on IL18 signaling (Kaplanski 2018; Hedl et al. 2014). This so-called IL18-IL18RAP axis has been found to make a big difference in cancers (Lin et al. 2020). One study suggested that IL18RAP was helpful in tumorigenesis of NKT cell lymphoma. In this disease, the protein encoded by this gene could activate NKT cell, while knockdown of this gene focus could reduce the proliferation of NKT cancer cell by arrest at G0/G1 and G2/M phases. This is a strong evidence of association between IL18RAP and NKT cell lymphoma (*P* < 1.0 × 10^−6^). Another study found that individuals carrying IL18RAP single-nucleotide polymorphisms rs917997 were more vulnerable to gastric cancer or precancerous disease (odds ratio = 1.83) (Wang et al. 2016). The study also mentioned that this gene polymorphism was significantly associated with ulcerative colitis and Crohn’s disease, which indicated the pro-inflammatory role of IL18RAP in these inflammatory gastrointestinal morbidities (Wang et al. 2016; Zhernakova et al. 2008). However, whether this gene affected other types of solid malignancies were not clear. Since HCC usually developed on the basis of chronic liver inflammation, IL18RAP has a high likelihood to play a key role in microenvironment of HCC.

Although these two genes have a significant clinical meaning, merely KLRB1 was illustrated in top 30 core genes associated with CD4^+^ T cells according to the PPI networks. IL18RAP was not illustrated either in PPI network of core genes associated with CD4^+^ T cells or CD8^+^ T cells. It is likely that there is no direct link between gene interaction and prognosis impact of genes. The former one is the issue related with molecular signaling pathways, whereas the latter one emphasizes on the relationship between genes’ expression and patients’ survival. The same was occurred in network of miRNA–target gene network. Still, we would like to further explore the potential signal pathways of these genes, especially related regulatory molecules, such as miRNA.

The other four genes related either to CD4^+^ or CD8 T cells are also pivotal in varieties of biochemical reactions. GPR182, short for G Protein-Coupled Receptor 182, is a member of G protein-coupled receptors (Kapas et al. 1995). It is highly expressed in tumor endothelial cells of zebrafish and mouse (Xiao et al. 2014). Especially, this gene is enriched in myeloid leukemia of zebrafish, suggesting that GPR182 plays a role in hematopoiesis no matter in healthy people or patients (Alghisi et al. 2013; Kwon et al. 2020). What’s more, one study found that GPR182 was produced in varieties of lymphatic endothelial cells that distributed in lymph nodes, skin, and tissue of intestine (Le Mercier et al. 2021). SPOCD1, abbreviation of “spen paralogue and orthologue C-terminal domain containing 1,” is a brand-new gene expressing a kind of the transcription factor S-II family (Kimura et al. 2006). At the beginning, this molecule was found to combine with testis protein phosphatase 1, a eukaryotic serine/threonine-specific phosphatase regulating cell signaling (Fardilha et al. 2011). Liu et al. found that SPOCD1 was significantly up-regulated in ovarian cancer and was correlated with a higher tumor grade and staging. The possible mechanism is activating PI3K/AKT pathway which slows down the apoptosis of ovarian cancer cells (Liu et al. 2020). Liang et al. suggested that this molecule, as a pro-oncogenic factor, was highly expressed in osteosarcoma tissue, especially the highly invasive phenotype (Liang et al. 2018). Some other studies also demonstrated that SPOCD1 was highly expressed bladder cancer and gastric cancer, stimulating cell proliferation, migration, and invasiveness (van der Heijden et al. 2016; Zhu et al. 2017). As for IL7R, interleukin-7 receptor, it plays an important role in tumor immunity through the conventional JAK/STAT pathway as well as endogenous competitive regulating RNA networks (Fan et al. 2018). A study illustrated that it is significantly related to immune cells, including CD8 T cell and Treg and somatic mutations (He et al. 2020a, b). This conclusion was also supported by Yin’s research, which said that high expression of IL7R can enhance anti-tumor immunity and indicate a better prognosis of HCC patients (Yin et al. 2020).

Despite some novel findings in our study, some limitations have to be noticed: (1) for Cibersort algorithm, HCC samples eligible for analysis of abundance of immune cells were no more than 100 and a larger sample size of eligible samples for Cibersort algorithm for data integration of immune cells is necessary; (2) due to heterogeneous molecular phenotypes of regulatory T cells, we cannot put this subgroup of T cells in either CD4^+^ or CD8^+^ T cell to complete further analysis and the relationship of relative abundance of regulatory T cells and genes cannot be clarified in our study; (3) since survival analysis is a clinical issue, the association between the mentioned genes and the prognosis of HCC patients is necessary to be proved in future clinical trials; and (4) down-regulated genes in PPI and miRNA–target gene network will be encouraged to explore in future investigation.

## Conclusion

To conclude, DEGs between high and low stromal/immune scores were determined. Genes related to tumor infiltration T cells were also be identified. Among, a total of six genes related to CD4^+^ and/ or CD8^+^ T cells had a significant impact on OS of HCC patients, in which KLRB1 and IL18RAP were two genes related to both of these two types of immune cells in HCC samples.

## Supplementary Information

Below is the link to the electronic supplementary material.

Supplementary file1 (DOCX 2112 kb)

Supplementary file2 (DOCX 27 kb)

## Data Availability

The Cancer Genome Atlas Hepatocellular Carcinoma (TCGA-LIHC) data collection were downloaded from TCGA GDC website (https://portal.gdc.cancer.gov/); data of stromal and immune score were using ESTIMATE algorithm, which are originated from officiate website of ESTIMATE (https://bioinformatics.mdanderson.org/estimate/); archives of gene symbols were downloaded from Ensemble genome browser 103 (http://ftp.ensembl.org/pub/current_gtf/homo_sapiens/); Cibersort algorithm and its Appendix reference data from official website of CIBERSORTX (https://cibersortx.stanford.edu/). The raw materials of PPI network and miRNA–target gene networks were produced via official websites of STRING (https://www.string-db.org/) and miRwalk 3.0 (http://mirwalk.umm.uni-heidelberg.de/).

## Abbreviations

BP: Biological process
CC: Cellular component
CTLA-4: Cytotoxic T-lymphocyte-associated protein 4
DEGs: Differentiated expressed genes
ESTIMATE: Estimation of Stromal and Immune cells in Malignant Tumor tissues using Expression data
GO: Gene Ontology
HCC: Hepatocellular carcinoma
HR: Hazard ratio
IFN-γ: Interferon-γ
KEGG: Kyoto Encyclopedia of Genes and Genomes
K–M: Kaplan–Meier
logFC: Logarithm of fold changes of genes
MF: Molecular function
miRNA: MicroRNA
NK cell: Natural killer cell
OS: Overall survival
PD-1: Programmed cell death protein 1
PLC: Primary liver cancer
PPI: Protein–protein interaction
TCGA: The Caner Genome Atlas
TIL: Tumor-infiltrating lymphocytes
TME: Tumor microenvironment
