# Supplementary material for: Key Genes Associated with Tumor-Infiltrating Non-regulatory CD4- and CD8-Positive T Cells in Microenvironment of Hepatocellular Carcinoma
Source: Biochem Genet. 2022 Jan 29;60(5):1762–80. doi: 10.1007/s10528-021-10175-3 (PMC9470630; doi:10.1007/s10528-021-10175-3)
Supplement: Supplementary file 1 — Supplementary file1 (DOCX 2112 kb) [file 10528_2021_10175_MOESM1_ESM.docx]

Key genes associated with tumor-infiltrating non-regulatory CD4 and CD8 positive T cells in microenvironment of hepatocellular carcinoma

Zijun Zhao^1^ Chaonan Wang^2^ Peishan Chu^3^ Xin Lu^1^

1 Department of Liver Surgery, Peking Union Medical College Hospital, Chinese Academy of Medical Sciences and Peking Union Medical College, Beijing, China.

2 Department of Vascular Surgery, Peking Union Medical College Hospital, Chinese Academy of Medical Sciences and Peking Union Medical College, Beijing, China.

3 Department of Cardiac Surgery, Peking Union Medical College Hospital, Chinese Academy of Medical Sciences and Peking Union Medical College, Beijing, China.

Corresponding author: Xin Lu, MD, Professor, Department of Liver Surgery, Peking Union Medical College Hospital, Chinese Academy of Medical Sciences and Peking Union Medical College, 1 Shuaifuyuan, Wangfujing, Beijing 100730, China. luxinln@163.com

**Supplementary Fig.1 Heatmap of top 50 upregulated and downregulated DEGs based on stromal score in HCC samples**

DEGs, differential expressed genes; HCC, hepatocellular carcinoma

**Supplementary Fig.2 Heatmap of top 50 upregulated and downregulated DEGs based on immune score in HCC samples**

DEGs, differential expressed genes; HCC, hepatocellular carcinoma


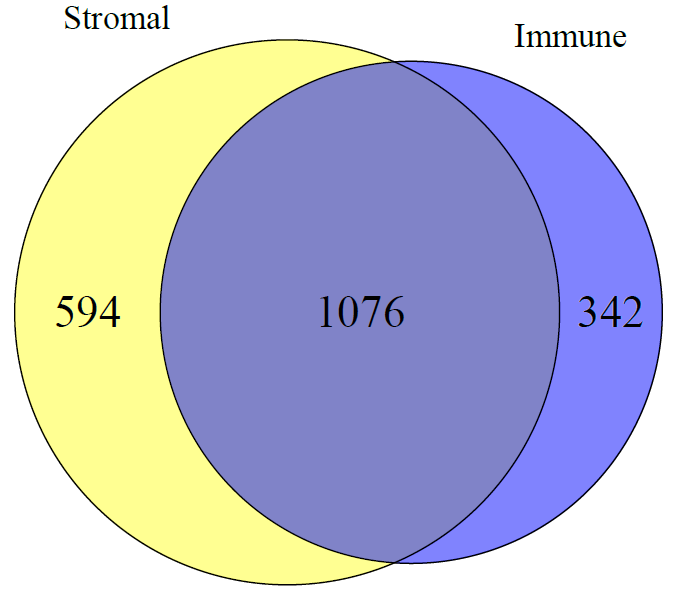
(a)


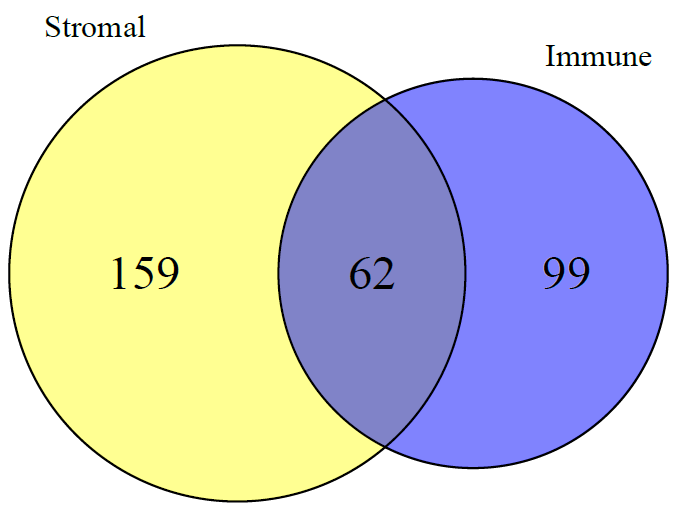
(b)

**Supplementary Fig.3 Venn diagram of overlapping upregulated (a) and downregulated (b) DEGs in HCC samples based on stromal and immune scores**

DEGs, differentially expressed genes; HCC, hepatocellular carcinoma

(a)

(b)

**Supplementary Fig.4 The bar-plot (a) and bubble plot (b) of GO analysis of overlapping genes of DEGs**

BP, biological process; CC, cellular component; DEGs, differentiated expressed genes; MF, molecular functions; GO, Gene Ontology

(a)

(b)

**Supplementary Fig.5 The bar-plot (a) and bubble plot (b) of KEGG analysis for overlapping DEGs**

BP, biological process; CC, cellular component; DEGs, differentiated expressed genes, KEGG, Kyoto Encyclopedia of Genes and Genomes; MF, molecular functions

**Supplementary Fig.6 Bar-plot showed the infiltration abundance ratio of major immune cell subtypes in 77 eligible samples (P<0.05).**


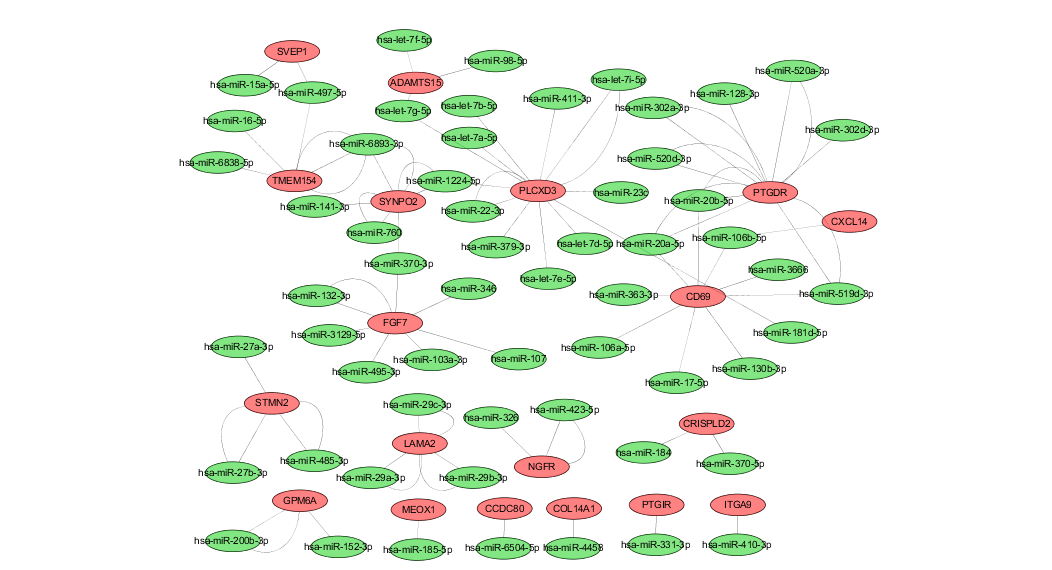
(a)


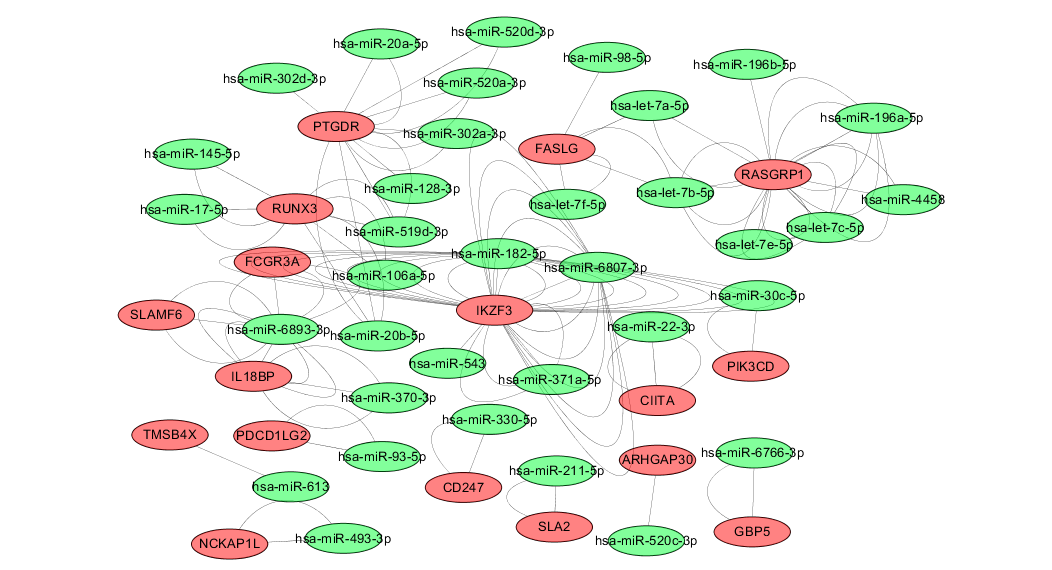
(b)

**Supplementary Fig.7 mi-RNA-target gene interaction networks. The miRNA network of CD4^+^ T cell-related genes (a) and CD8^+^ T cells (b). Red nodes represent T cell-related genes while green nodes represented miRNAs.**

miRNA, microRNA


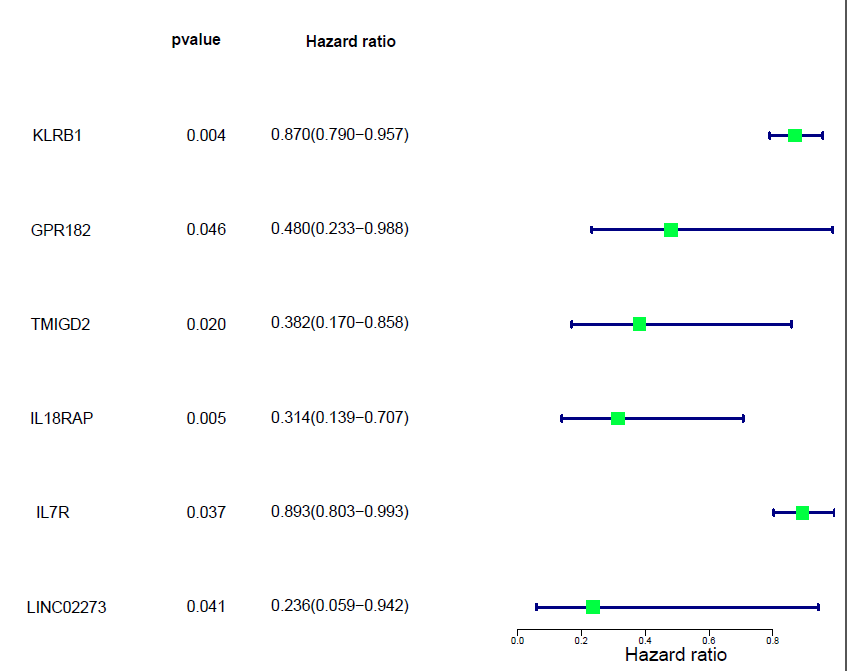
(a)


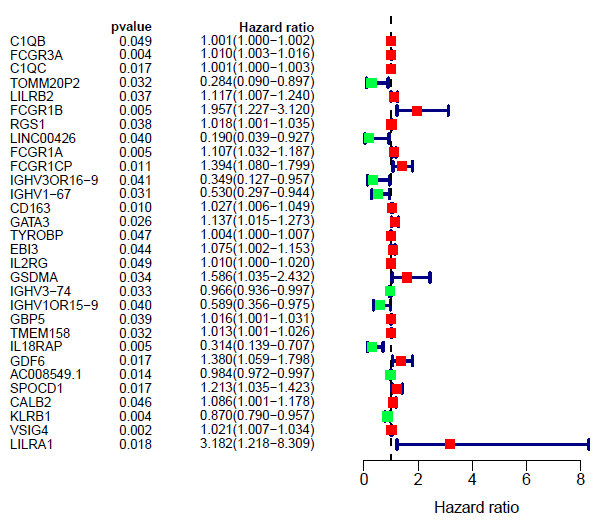
(b)

**Supplementary Fig.8 Forest plots of significantly prognosis (OS)-related genes associated with CD4 (a) and CD8 (b) positive T cells in HCC patients. The Figure 8A demonstrated six genes and all of them were labeled as green, which referred as these genes were beneficial for survival of HCC patients (HR<1) and Figure 8B illustrated 30 genes and 21 of them were high-risk genes labeled with red color, meaning that their high expression suggested a poor prognosis and a short OS (HR>1)**

HCC, hepatocellular carcinoma; OS, overall survival

(a) (b)

(c) (d)

**Supplementary Fig.9 Other 4 Genes significantly associated with prognosis of HCC patients. Among these genes, GPR182 (A) and IL7R (B) were genes related to CD4^+^ T cells while SPOCD1 (c) and LINC00426 (d) were two genes related to CD8^+^ T cells.**

HCC, hepatocellular carcinoma
