# Supplementary material for: Key Genes Associated with Tumor-Infiltrating Non-regulatory CD4- and CD8-Positive T Cells in Microenvironment of Hepatocellular Carcinoma
Source: Biochem Genet. 2022 Jan 29;60(5):1762–80. doi: 10.1007/s10528-021-10175-3 (PMC9470630; doi:10.1007/s10528-021-10175-3)
Supplement: Supplementary file 2 — Supplementary file2 (DOCX 27 kb) [file 10528_2021_10175_MOESM2_ESM.docx]

Key genes associated with tumor-infiltrating non-regulatory CD4 and CD8 positive T cells in microenvironment of hepatocellular carcinoma

Zijun Zhao^1^ Chaonan Wang^2^ Peishan Chu^3^ Xin Lu^1^

1 Department of Liver Surgery, Peking Union Medical College Hospital, Chinese Academy of Medical Sciences and Peking Union Medical College, Beijing, China.

2 Department of Vascular Surgery, Peking Union Medical College Hospital, Chinese Academy of Medical Sciences and Peking Union Medical College, Beijing, China.

3 Department of Cardiac Surgery, Peking Union Medical College Hospital, Chinese Academy of Medical Sciences and Peking Union Medical College, Beijing, China.

Corresponding author: Xin Lu, MD, Professor, Department of Liver Surgery, Peking Union Medical College Hospital, Chinese Academy of Medical Sciences and Peking Union Medical College, 1 Shuaifuyuan, Wangfujing, Beijing 100730, China. luxinln@163.com

**Supplementary Table.1 Top 50 upregulated and downregulated genes according to stromal scores in HCC samples**

| **Upregulated gene** | **logFC** | **Downregulated gene** | **logFC** |
| --- | --- | --- | --- |
| TRARG1 | 8.082049066 | STRC | -6.335848074 |
| CRTAC1 | 5.921075388 | RNU6ATAC | -5.645959835 |
| MUCL3 | 5.823612095 | PROK1 | -3.509361786 |
| IGFN1 | 5.717135723 | CPNE6 | -3.173288757 |
| TRIM63 | 5.215684123 | PAGE1 | -2.980471633 |
| C16orf89 | 4.967008887 | FOXH1 | -2.856879957 |
| DAPL1 | 4.947872953 | REG4 | -2.841542898 |
| GDF2 | 4.891569929 | PAGE2B | -2.795325765 |
| PLPP4 | 4.500337614 | STRCP1 | -2.556259527 |
| IGKV2D-40 | 4.487509829 | KLK4 | -2.505939529 |
| IGLV10-54 | 4.419587605 | FGF17 | -2.436146641 |
| COL11A1 | 4.351022583 | TBX4 | -2.374778583 |
| WNT2 | 4.288318421 | RGSL1 | -2.370476253 |
| OMG | 4.258833819 | DLX1 | -2.308453521 |
| CCL19 | 4.238587431 | CYP1A1 | -2.286445108 |
| IL11 | 4.173026034 | AC005550.2 | -2.249682981 |
| IGLV5-45 | 4.008484507 | RHBDL3 | -2.224201411 |
| IGKV6-21 | 3.947455429 | LINC02587 | -2.181214627 |
| IGKV1D-12 | 3.9470815 | CTSV | -2.173431007 |
| CLEC1B | 3.930779893 | PNCK | -2.077889774 |
| COL10A1 | 3.878675149 | AC104088.1 | -2.065658348 |
| IGHV1-58 | 3.856422113 | LINC01139 | -1.981241083 |
| IGKV1D-42 | 3.849714629 | CASC22 | -1.964291329 |
| CR2 | 3.779717946 | LINC02413 | -1.949064322 |
| CHIT1 | 3.674546839 | MYH7B | -1.932967117 |
| IGLV2-8 | 3.660872972 | AL355987.4 | -1.909625906 |
| LUM | 3.651212328 | AURKBP1 | -1.899215863 |
| SCARA5 | 3.645276634 | AC007277.1 | -1.893492846 |
| TNNT3 | 3.636023895 | RASL10B | -1.881206065 |
| CHRNA1 | 3.629784633 | AC109454.1 | -1.866778185 |
| CCDC80 | 3.585411999 | CACNG4 | -1.842834995 |
| ISLR | 3.567625948 | AC026765.3 | -1.836960052 |
| CCL21 | 3.562573732 | CTNNA2 | -1.803218139 |
| DES | 3.54427518 | RHBG | -1.797596573 |
| HAND2 | 3.488497059 | PGC | -1.792582727 |
| NDNF | 3.486649468 | AC239809.3 | -1.759106346 |
| IGHV3-11 | 3.48532103 | AC079305.1 | -1.753238308 |
| OMD | 3.472906791 | GLULP4 | -1.74924758 |
| CRYBB1 | 3.45899912 | TAC3 | -1.740084769 |
| DCN | 3.449092866 | NOTUM | -1.721614096 |
| PLN | 3.418497157 | AC011747.1 | -1.705693395 |
| PDPN | 3.387490547 | LINC02241 | -1.701313057 |
| LRRC15 | 3.356660872 | LINC01970 | -1.682675859 |
| IGLC7 | 3.352615496 | AL163953.1 | -1.675804562 |
| OGN | 3.349088748 | AL590483.2 | -1.670323884 |
| STMN2 | 3.337090583 | AC006205.2 | -1.652998531 |
| KLK10 | 3.319065432 | VCX | -1.621779393 |
| FNDC1 | 3.310352966 | RPL10L | -1.602827232 |
| HAND2-AS1 | 3.307586928 | PAGE4 | -1.558153316 |
| SHISA3 | 3.304890545 | MIR325HG | -1.536526543 |

FC, fold change; HCC, hepatocellular carcinoma

**Supplementary Table.2 Top 50 upregulated and downregulated genes according to immune scores in HCC samples**

| **Upregulated gene** | **logFC** | **Downregulated gene** | **logFC** |
| --- | --- | --- | --- |
| TRARG1 | 7.250060392 | KLK4 | -3.275140313 |
| OLFM4 | 5.835701463 | KCNH6 | -3.183692673 |
| IGKV1D-42 | 5.801011683 | SH3GL3 | -2.713826389 |
| IGKV1D-17 | 5.70791511 | AP000593.3 | -2.643884682 |
| IGLV3-27 | 5.536321141 | HMGN2P40 | -2.486272842 |
| IGHV3-73 | 5.228248029 | ANKFN1 | -2.395505435 |
| CRTAC1 | 5.144295018 | B3GALT1 | -2.32401705 |
| IGHV1-58 | 5.026042773 | C1QTNF3 | -2.301594753 |
| IGLV3-9 | 5.015896563 | SLC6A2 | -2.204226608 |
| IGLV5-45 | 5.015809762 | AQP6 | -2.151685298 |
| IGKV1-9 | 4.937247629 | GRPR | -2.1499686 |
| IGKV6-21 | 4.915918153 | RHBDL3 | -2.144257622 |
| IGLV4-69 | 4.840506802 | LINC02587 | -2.107043069 |
| IGLV3-19 | 4.823804921 | AL590483.2 | -2.092385601 |
| IGFN1 | 4.812896945 | AVIL | -2.087389367 |
| IGHV1-2 | 4.79821952 | KCNU1 | -2.061641034 |
| IGLV3-16 | 4.796296464 | RGSL1 | -2.040403959 |
| C16orf89 | 4.764736336 | NKD1 | -2.027913632 |
| IGHV3OR16-8 | 4.757107796 | PAGE4 | -1.998567776 |
| IGHV3-66 | 4.702209608 | PCP4 | -1.958941083 |
| IGKV3D-20 | 4.693544046 | SULT4A1 | -1.952185971 |
| IGHV4-39 | 4.631813972 | ACTN2 | -1.945663469 |
| LINC01819 | 4.62016466 | DRD1 | -1.925130826 |
| IGLV3-10 | 4.608711489 | AURKBP1 | -1.890140559 |
| IGKV1D-13 | 4.595098154 | AC007277.1 | -1.889402541 |
| IGLV8-61 | 4.585940198 | HNRNPA1P66 | -1.808955909 |
| IGHV3-53 | 4.573850531 | UGT1A2P | -1.801069351 |
| IGKV6D-21 | 4.545162877 | CYP3A4 | -1.790236592 |
| IGHV3-64 | 4.53617301 | RHBG | -1.763745571 |
| IGHV3-49 | 4.529648707 | FAM240C | -1.727222816 |
| PLA2G2D | 4.496948631 | RASL10B | -1.702045944 |
| IGHV4-4 | 4.4888816 | UGT1A3 | -1.682900963 |
| IGLV10-54 | 4.461872666 | LRRC52 | -1.662583493 |
| IGHV4-31 | 4.412346863 | ABHD1 | -1.642657567 |
| IGKV2D-40 | 4.376991125 | LGR5 | -1.637818666 |
| IGLV3-12 | 4.346650593 | AC069294.1 | -1.637151024 |
| IGLV3-1 | 4.342099113 | CTNNA2 | -1.631904548 |
| IGKV3-15 | 4.34207966 | CYP2A13 | -1.625210017 |
| IGLV3-25 | 4.309321674 | ACSL6 | -1.6032429 |
| IGKV3D-15 | 4.307425758 | SPATA46 | -1.596677188 |
| IGLV2-23 | 4.273626159 | GDNF-AS1 | -1.59582077 |
| IGHV3-43 | 4.259753476 | MTND4P35 | -1.561575215 |
| IGLV2-11 | 4.25308177 | AC026765.3 | -1.53379004 |
| IGKV3D-11 | 4.237743756 | ASS1P2 | -1.517936211 |
| IGKV3-11 | 4.22069581 | TERB2 | -1.48454732 |
| IGHV1-3 | 4.175979592 | AXIN2 | -1.478469064 |
| IGHV3-33 | 4.173660361 | GLUL | -1.472130201 |
| IGHV3-23 | 4.141633326 | AC011747.1 | -1.464715315 |
| IGLV4-60 | 4.110540628 | CNNM1 | -1.458701664 |
| IGHV3-15 | 4.102818133 | BX470209.1 | -1.442812338 |

FC, fold change; HCC, hepatocellular carcinoma
